# The complete chloroplast genome of *Tinospora sinensis* and its phylogenetic analysis

**DOI:** 10.1080/23802359.2025.2582534

**Published:** 2025-11-09

**Authors:** Jiahua Chen, Yang Ni, Guoan Shen, Chang Liu

**Affiliations:** ^a^ School of Chemistry and Chemical Engineering, Guangdong Pharmaceutical University, Guangzhou, Guangdong, P. R. China; ^b^ Institute of Medicinal Plant Development, Chinese Academy of Medical Sciences, Peking Union Medical College, Beijing, P. R. China

**Keywords:** *Tinospora sinensis*, chloroplast genome, phylogenetic analysis

## Abstract

*Tinospora sinensis* (Lour.) Merr. (Menispermaceae) is a medicinal plant with limited genomic resources available. Here, we assembled and annotated its 160,664 bp chloroplast genome, identifying 126 genes with a GC content of 39.31%. We detected 256 SSRs and 50 dispersed repeats, which represent potential molecular markers. IR boundary analysis showed slight expansions/contractions involving *rps19*, *ndhF*, and *ycf1*. Phylogenetic analysis confirmed its close evolutionary relationship with other *Tinospora* species. This is the first comprehensive genomic characterization of *T. sinensis*, providing valuable resources for taxonomy, evolution, and medicinal research in Menispermaceae.

## Introduction

*Tinospora sinensis* (Lour.) Merr. 1934 also known as *Qingjiuniu* in Chinese, is a climbing plant of the genus *Tinospora* of the family Menispermaceae. It is widely distributed across India, China, Northwestern South Africa, Myanmar, and Sri Lanka. Previous studies have revealed close genetic similarities among the three *Tinospora* species found in India: *T. sinensis*, *T. cordifolia*, and *T. crispa* (Liang et al. 2024). The stems of *T. sinensis* are commonly used as traditional herbal medicines. Recent studies have identified 57 chemical compounds from *T. sinensis*, including three newly identified structures belonging to lignans, alkaloids, and phenolic compounds (Lam et al. 2018; Liang et al. 2024). Pharmacological investigations have demonstrated its antioxidant, anti-neuroinflammatory, and antidiabetic properties (Banerjee et al. 2020; Zhou et al. 2020). In addition, *Tinospora* species are widely used as components in various traditional formulations (Upadhyay et al. 2010; Lam et al. 2012). While a chloroplast genome sequence (MN727386) of *T. sinensis* is available in GenBank of NCBI, the absence of a formal publication and detailed characterization limits its utility. Therefore, there is an urgent need to clarify the phylogenetic relationships of *T. sinensis* and its closely related species, and to identify reliable molecular markers to support bioprospecting and quality assurance of *T. sinensis* products. This study provides the first comprehensive analysis of the *T. sinensis* chloroplast genome to address these gaps.

Chloroplasts are double-membraned organelles found in plants, algae, and certain protists. In green plants, they serve as the primary organelles for photosynthesis and possess their own genome. Chloroplast genomes typically range from 120 to 160 kb in size and exhibit a highly conserved quadripartite structure (Lu et al. 2023; Zhang et al. 2023). The chloroplast genomes consist of a single circular DNA molecule composed of two inverted repeat (IR) regions, which separate the large single-copy (LSC) region from the small single-copy (SSC) region (Daniell et al. 2016). With the advancement of high-throughput sequencing technologies, chloroplast genomes have been increasingly applied in studying plant systematics and taxonomy. They have become important molecular tools for investigating plant genetic diversity and evolutionary relationships. To date, one *T. sinensis* chloroplast genome sequence has been submitted to the public database (MN727386). However, no formal publication associated with it could be found.

In this study, we assembled and annotated the complete chloroplast genome of *T. sinensis* using high-throughput sequencing technology, aiming to characterize its structural features and determine the phylogenetic relationship of *T. sinensis* and its congeneric species. This research provides essential molecular data to support the conservation and utilization of *T. sinensis*, and contributes valuable genomic resources for the phylogenetic and evolutionary study of Menispermaceae.

## Materials and methods

**Figure 1.**
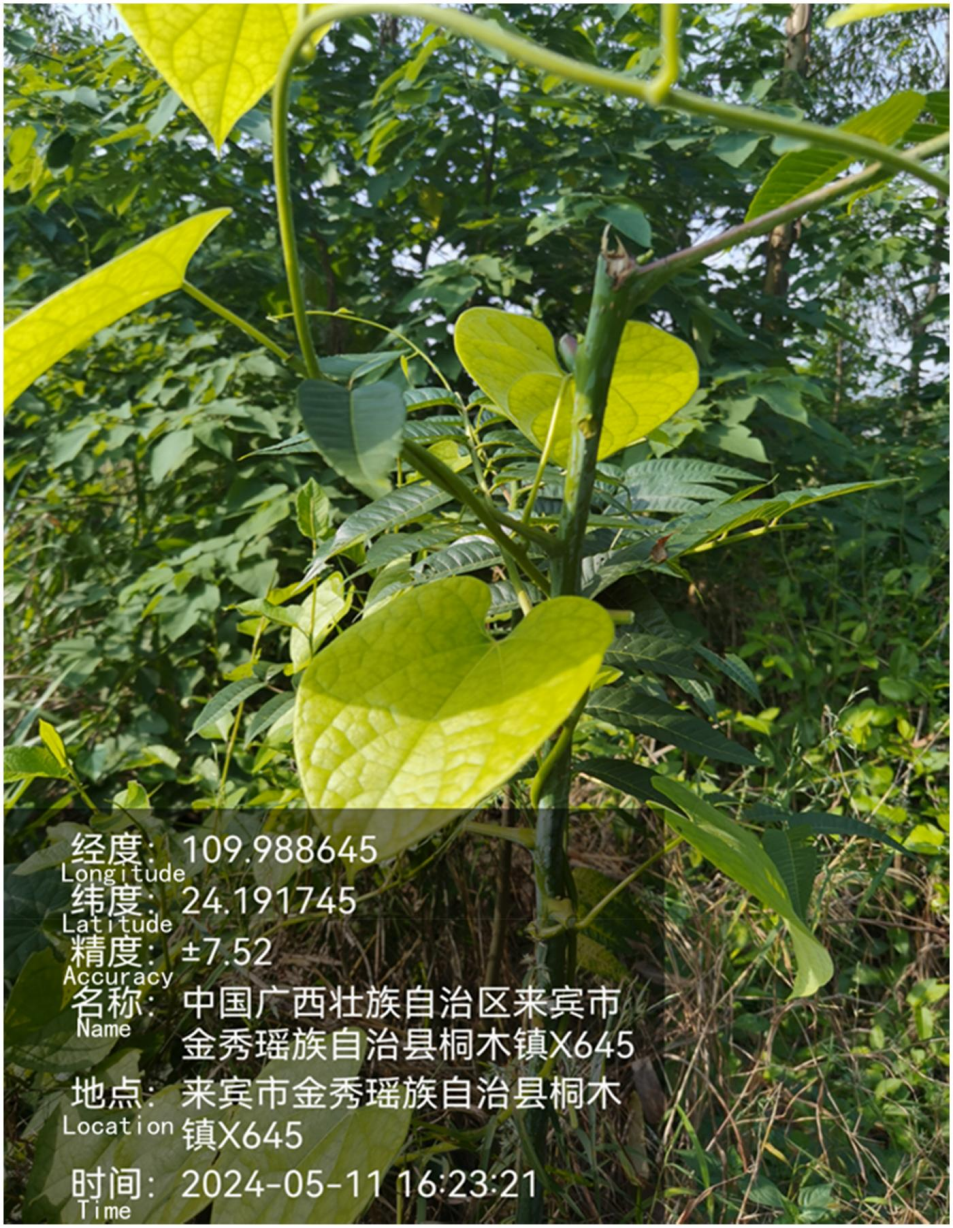
The leaves of *T. sinensis*, photographed by Jiahua Chen in Jinxiu Yao Autonomous County, Guangxi, China. The collection information, added automatically at the time of photographing, are shown in the left lower corner. The translation was shown in square bracket. The features of the plant are the following: leaves broadly ovate to suborbicular, entire-margined, apex acute, base deeply cordate or slightly cordate, both surfaces covered with soft hairs.

Fresh leaves from a single individual were collected from Tongmu Township, Jinxiu Yao Autonomous County, Laibin City, Guangxi Zhuang Autonomous Region, China (latitude 24.191745°, longitude 109.988645°; altitude: 130 m) and immediately preserved in liquid nitrogen. Sampling was authorized by Xinmin Pan (40971972@qq.com). The voucher specimen (No. JXHC015) has been deposited in the herbarium of the Medicinal Plant Garden, Institute of Medicinal Plant Development (Figure 1).

Total DNA was extracted using the Plant Genomic DNA Kit, whole-genome sequencing was performed on the PacBio Sequel II platform, generating ∼10 Gb of high-fidelity (HiFi) long-read data. The long-read data were used for de novo assembly of the complete plastid genome using the oatk assembler (v1.0) (Kolmogorov et al. 2019).

To verify the structural integrity and accuracy of the assembly, the resulting genome was visualized using Bandage v0.8.1 (Wick et al. 2015). In addition, we mapped the reads to the assemblies to determine how well the assemblies were supported by the reads (Ni et al. 2023). The genome was annotated using GeSeq (Tillich et al. 2017) and CPGAVAS2 (Shi et al. 2019). The correctness of the annotation results was examined using CPGView (Liu et al. 2023). All genomic features, including cis-spliced and trans-spliced genes were also visualized by CPGview, whereas circular genome mapping was generated by the GeSeq online website tool (https://chlorobox.mpimp-golm.mpg.de/geseq.html).

To quantitatively evaluate the preferential usage of individual codons among synonymous alternatives, the Relative Synonymous Codon Usage (RSCU) metric has been widely adopted in codon usage studies. In this study, RSCU values were calculated using the following formula (Choudhuri and Sau 2024):

$${\text{RSCU}}_{\text{ij}}=\frac{x_{\text{ij}}}{\frac{1}{n_{i}}\sum_{j=1}^{n_{i}}x_{\text{ij}}}$$

Interspersed repeat sequences were identified using the online tool REPuter (https://bibiserv.cebitec.uni-bielefeld.de/reputer), with the parameters set to a maximum of 50 repeats and a minimum repeat size of 8 bp (Kurtz et al. 2001). Four types of repeats were detected: forward, palindromic, reverse, and complement repeats. Simple sequence repeats (SSRs), also known as microsatellites, are short tandemly repeated DNA sequences widely distributed throughout the genome. They typically consist of repeat motifs ranging from 2 to 6 nucleotides in length, repeated in tandem multiple times (Alves et al. 2023). MISA (Microsatellite Identification Tool) was used for the prediction of SSRs (Beier et al. 2017), with parameter settings as follows: copy numbers of single-base repeats > 8, double-base repeats > 5, triple-base repeats > 3, quadruple-base repeats > 3, quintuple-base repeats > 3, and hexa-base repeats > 3.

To visualize the junctions between the inverted repeat (IR), large single-copy (LSC), and small single-copy (SSC) regions of the chloroplast genomes, we employed IRscope (Amiryousefi et al. 2018).

For phylogenetic analysis, complete chloroplast or plastid genome sequences of related species from different genera of the same family were downloaded from the GenBank database and the target species were processed using PhyloSuite (v1.2.3) (Zhang et al. 2019). The conserved genes common to all species were extracted using PhyloSuite and the maximum likelihood phylogenetic tree was reconstructed by running UFBS (UltraFast BootStraps) 1000 times using IQ-TREE and cpREV substitution models. The generated trees were visualized using the Interactive Tree of Life (iTOL) platform (https://itol.embl.de/) (Letunic and Bork 2024).

## Results

We obtained ∼21 Gb of raw sequencing data. The length of the assembled chloroplast genome was 160,664 bp, and the coverage depth ranged from a minimum of 128× to a maximum of 4405× (Figure S1) suggesting that the assembly is strongly supported with the sequencing reads. The high sequencing depth and coverage indicate that the genome assembly is highly reliable and suitable for subsequent structural and comparative analyses.

The plastome has a typical quadripartite structure, including a large single-copy (LSC) region of 92,353 bp, a small single-copy (SSC) region of 20,435 bp, and two inverted repeats (IR) regions of 23,938 bp each. The average GC content was 39.31%.

**Figure 2.**
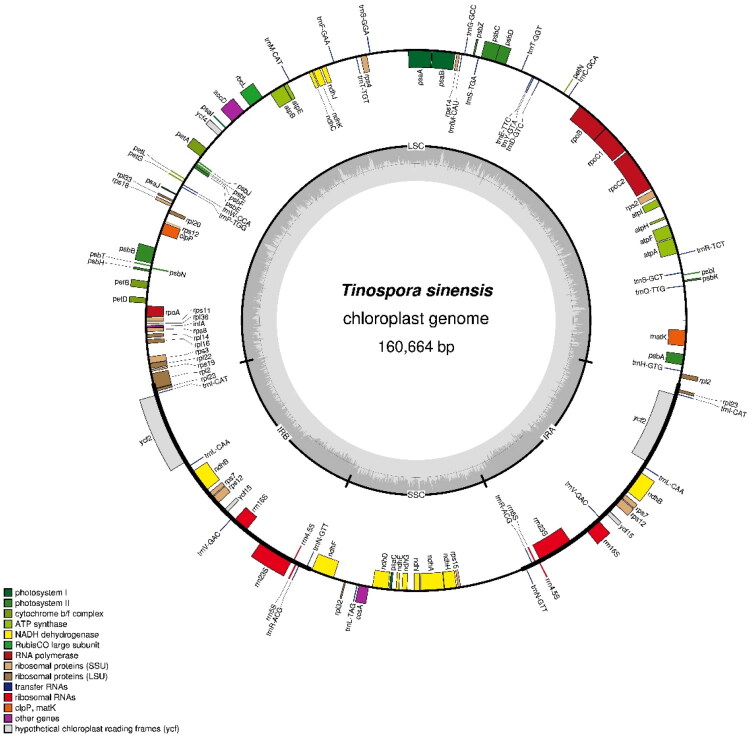
Circular gene map of the chloroplast genome of *T. sinensis*. The genome has a typical quadripartite structure, consisting of a large single-copy (LSC) region, a small single-copy (SSC) region, and a pair of inverted repeats (IRA and IRB), totaling 160,664 bp. Genes are color-coded according to functional categories, including genes involved in photosynthesis (photosystem I and II, ATP synthase, cytochrome b6f complex, and NADH dehydrogenase), transcription and translation (RNA polymerase, ribosomal proteins, rRNAs, tRNAs), and other functions. Genes transcribed clockwise are located on the outer side, and those transcribed counterclockwise are on the inner side of the circle.

A total of 126 functional genes are annotated, including 82 protein-coding genes (PCGs), 36 transfer RNA (tRNA) genes, and 8 ribosomal RNA (rRNA) genes (Figure 2). A total of 9 PCGs [*atpF*, *rpoC1*, *petB*, *petD*, *rpl16*, *rpl2*, *ndhB* (2x), and *ndhA*] contained one intron, while 4 genes [*ycf3*, *clpP*, and *rps12* (2x)] contained two introns (Figure S2). In addition, 7 tRNA genes [*trnK-UUU*, *trnT-CGU*, *trnL-UAA*, *trnE-UUC* (2x), *trnA-UGC* (2x)] also contained one intron (Figure S2). Furthermore, *rps12* gene was trans-spliced and had three independent exons, two of which were duplicated in the IR region (Figure S3).

Then we estimated the relative synonymous codon usage (RSCU) and codon usage frequency. In the chloroplast genome of *T. sinensis*, a total of 64 codons were identified, including three stop codons. The codon–anticodon recognition pattern revealed that among the stop codons, UAA was the most frequently used. In addition, GCU for alanine, AGA for arginine, UUA for leucine, UCU for serine, and ACU for threonine exhibited relatively high usage frequencies (Figure S4).

Among all codons, 31 codons showed RSCU values >1, of which 29 (93.54%) ended with either A or U. Conversely, among the 31 codons with RSCU values <1, 28 (90.32%) ended with either C or G. These findings indicate a strong codon usage bias toward codons ending in A or U in this chloroplast genome.

Repeat sequence analysis using the REPuter software identified a total of 50 dispersed repeats, including 21 forward repeats, 7 reverse repeats, 19 palindromic repeats, and 3 inverted repeats.

Their distribution patterns provide insights into genome stability, sequence evolution, and potential functional roles, serving as a molecular basis for investigating genomic evolutionary mechanisms. The presence of numerous palindromic and forward repeats suggests a potential role in promoting sequence rearrangements and plastome evolution in *T. sinensis* (Figure S5).

In the chloroplast genome of *T. sinensis*, a total of 256 SSRs were identified. Among these, mononucleotide repeats were the most abundant (140, 54.69%), followed by trinucleotide repeats (85, 33.20%), tetranucleotide repeats (17, 6.64%), dinucleotide repeats (12, 4.69%), and a small number of pentanucleotide and hexanucleotide repeats (each 1, 0.39%). Most mononucleotide SSRs were composed of A/T motifs, consistent with the AT-rich nature of chloroplast genomes (Figure S6). The abundance and distribution of these SSRs provide valuable resources for future population genetic and evolutionary studies in *Tinospora* species.

Comparative analysis of the chloroplast genome between our newly assembled (PV747743) and the released one (MN727386.1) revealed high sequence similarity and conserved genome structure. Overall, the alignment covered nearly the entire genome with >99.9% identity, indicating strong conservation at the genomic level. Despite this overall conservation, structural variations were detected at the boundaries of the LSC, SSC, and IR regions, prompting a detailed comparison of junction features.

The boundaries of the LSC, SSC, and IR regions are analyzed by comparing the newly assembled chloroplast genome of *T. sinensis* (PV747734) with the reference genome from the GenBank database (MN727386). The lengths of the LSC, SSC, and IR regions differed between the two genomes, reflecting potential structural variation. Notably, the IR/LSC junction in *T. sinensis* (PV747734) involves the *rpl2* gene. Such boundary variation may affect the copy number or expression of *rpl2*, which could in turn influence chloroplast ribosomal function. The biological significance of this structural difference is further discussed in the Discussion section (Figure S7).

In the newly assembled genome, the *rpl2* gene was found spanning the LSC/IRb junction, with 1466 bp of the gene located upstream within the IRb region. The *ndhF* gene was situated 61 bp upstream of the SSC/IRb border, while *rpl23* was located 536 bp downstream of the IRa region.

In contrast, the reference genome (MN727386) exhibited different boundary arrangements. The *rps19* gene was positioned at the LSC/IRb junction, with 74 bp extending into the IRb region. The *ndhF* gene was 63 bp upstream of the SSC region, while *ycf1* was located at the SSC/IRa junction, extending 107 bp into the IRa region. Additionally, the *trnH* gene was found at the IRa/LSC junction, with 16 bp located upstream in the LSC region.

These boundary differences suggest structural divergence at the junction sites between LSC, IR, and SSC regions, which may result from evolutionary rearrangements within the chloroplast genomes of *T. sinensis* from different geographical populations.

**Figure 3.**
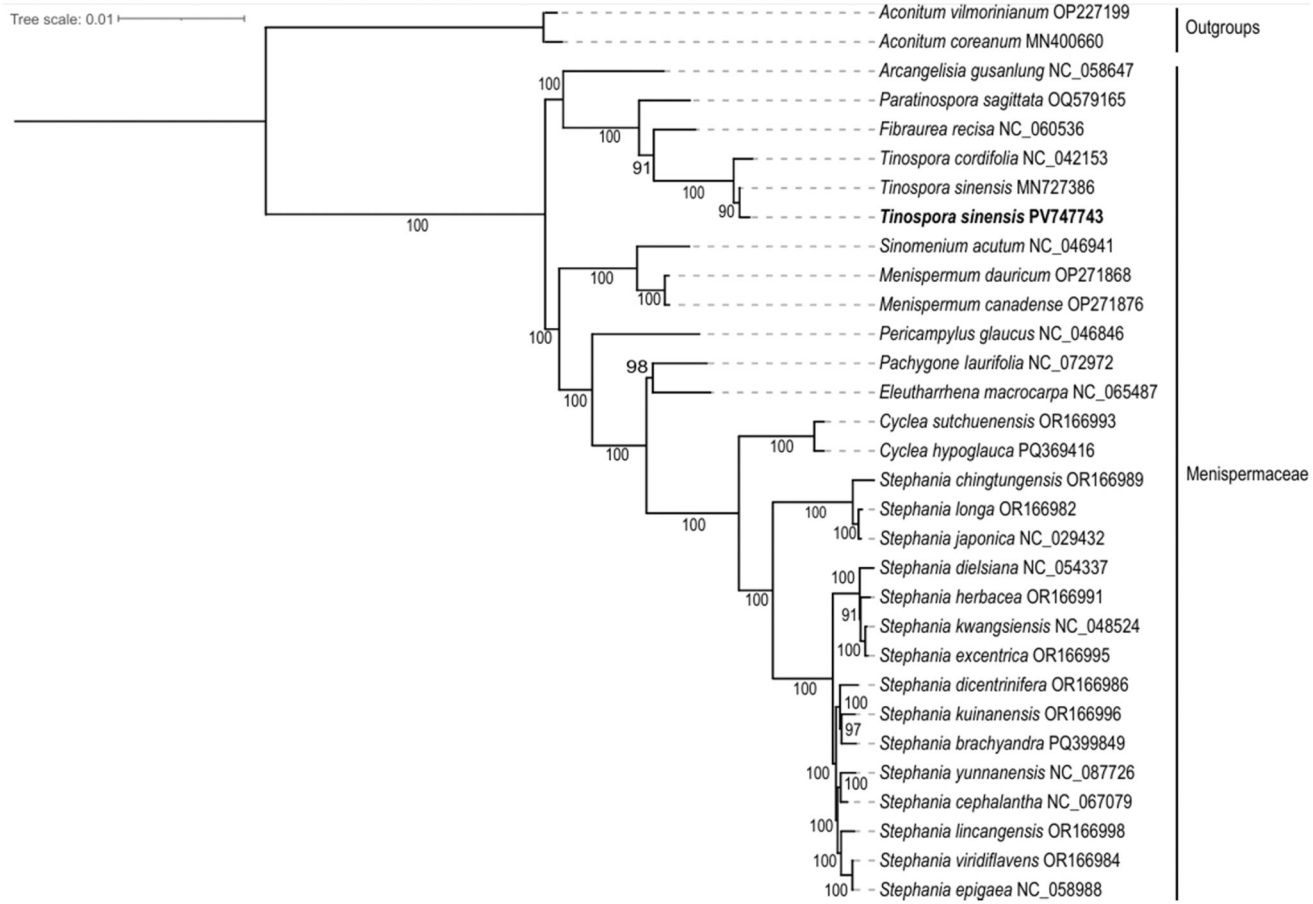
Phylogenetic tree of 29 Menispermaceae species inferred using the ML method based on 90 shared genes. *Aconitum coreanum* (MN400660.1) (Kim et al. 2019) and *Aconitum vilmorinianum* (OP227199.1) (unpublished) were selected as the outgroup. Numbers at the nodes represent the bootstrap values. The *T. sinensis* chloroplast genome obtained in this study (PV747743) is marked in bold. The sequences used for constructing the phylogenetic tree are as follows: *Arcangelisia gusanlung* (NC_058647.1) (Wen et al. 2021), *Paratinospora sagittata* (OQ579165.1) (unpublished), *Fibraurea recisa* (NC_060536.1) (Zheng and Feng 2022), *T. cordifolia* (NC_042153.1) (Monpara et al. 2024), *Tinospora sinensis* (MN727386.1) (direct submission), *Sinomenium acutum* (NC_046941.1) (Hina et al. 2020), *Menispermum dauricum* (OP271868.1) (direct submission), *Menispermum canadense* (OP271876.1) (direct submission), *Pericampylus glaucus* (NC_046846.1) (Kang and Wang 2019), *Pachygone laurifolia* (NC_072972) (direct submission), *Eleutharrhena macrocarpa* (NC_065487.1) (Song et al. 2022), *Cyclea sutchuenensis* (OR166993.1) (Wang et al. 2024), *Cyclea hypoglauca* (PQ369416.1) (direct submission), *Stephania chingtungensis* (OR166989.1) (Wang et al. 2024), *Stephania longa* (OR166982.1) (Wang et al. 2024), *Stephania japonica* (NC_029432.1) (Sun et al. 2016), *Stephania dielsiana* (NC_054337.1) (unpublished), *Stephania herbacea* (OR166991.1) (Wang et al. 2024), *Stephania kwangsiensis* (NC_048524) (Shi and Liu 2020), *Stephania excentrica* (OR166995.1) (Wang et al. 2024), *Stephania dicentrinifera* (OR166986.1) (Wang et al. 2024), *Stephania kuinanensis* (OR166996.1) (Wang et al. 2024), *Stephania brachyandra* (PQ399849.1) (Duong et al. 2025), *Stephania yunnanensis* (NC_087726.1) (direct submission), *Stephania cephalantha* (NC_067079.1) (Li et al. 2022), *Stephania lincangensis* (OR166998.1) (direct submission), *Stephania epigaea* (NC_058988.1) (direct submission), *Stephania viridiflavens* (OR166984.1) (Wang et al. 2024).

Phylogenetic analysis based on chloroplast protein-coding genes showed that our *T. sinensis* sequence was clustered with the *T. sinensis* sequence from GenBank (MN727386) with strong bootstrap support (90), and the two together formed a clade sister to *Tinospora cordifolia.* The phylogenetic tree also confirmed the monophyly of the *Tinospora* genus and clearly separated it from other related genera within Menispermaceae (Figure 3).

## Discussion and conclusion

In this study, we assembled and annotated the complete chloroplast genome of *T. sinensis*, a medicinally important species within the family Menispermaceae. The chloroplast genome exhibits a typical conserved quadripartite structure.

Previous taxonomic records have referred to *Tinospora sinensis* (Lour.) Merr. as a synonym of *T. cordifolia*, reflecting historical ambiguities in species delimitation (Sharma et al. 2019). Morphological comparisons reveal that although *T. sinensis* and *T. cordifolia* share several traits, such as broadly ovate leaves, pubescence on the abaxial surface, and robust stems, they differ in stem surface characteristics, fruit morphology, and growth habits (Pandey et al. 2020).

Variation in the boundaries of IR/LSC and IR/SSC regions is frequently observed across chloroplast genomes and plays a crucial role in genome size variation. In this study, the observed differences in the location and extent of genes, such as *rpl2*, *rps19*, *ndhF*, *ycf1*, and *trnH* at the junctions suggest lineage-specific expansions or contractions of the IR regions in *T. sinensis*. These boundary shifts, although minor, may contribute to genomic rearrangements and evolutionary divergence among different populations or ecotypes of the same species.

In summary, this study provides the first comprehensive comparative analysis of the *T. sinensis* chloroplast genome, going beyond simple genome assembly to reveal key structural and evolutionary features. The identification of IR boundary variation involving *rpl2* highlights potential lineage-specific structural divergence within *T. sinensis*, offering clues to chloroplast genome evolution in Menispermaceae. The detection of abundant SSRs and dispersed repeats provides valuable molecular markers for future studies on population genetics, species authentication, and resource conservation. Furthermore, phylogenetic reconstruction based on chloroplast protein-coding genes confirmed the monophyly of the *Tinospora* genus and resolved the placement of *T. sinensis* as a sister species to *T. cordifolia* with strong support. Collectively, these findings deepen our understanding of the genomic architecture and evolutionary relationships within *Tinospora* and establish a solid foundation for future taxonomic, phylogenomic, and medicinal studies.

## Supplementary Material

Supplemental Material

## Data Availability

The genome sequence data supporting this study are openly available in GenBank of NCBI at https://www.ncbi.nlm.nih.gov under the accession number PV747743. The associated BioProject, BioSample, and SRA numbers are PRJNA1272258, SAMN48903800, and SRR33838211, respectively.
