# Supplementary material for: The complete chloroplast genome of Tinospora sinensis and its phylogenetic analysis
Source: Mitochondrial DNA B Resour. 2025 Nov 9;10(12):1142–8. doi: 10.1080/23802359.2025.2582534 (PMC12599365; doi:10.1080/23802359.2025.2582534)
Supplement: Supplemental Material [file TMDN_A_2582534_SM9507.docx]

## **Supplement materials**


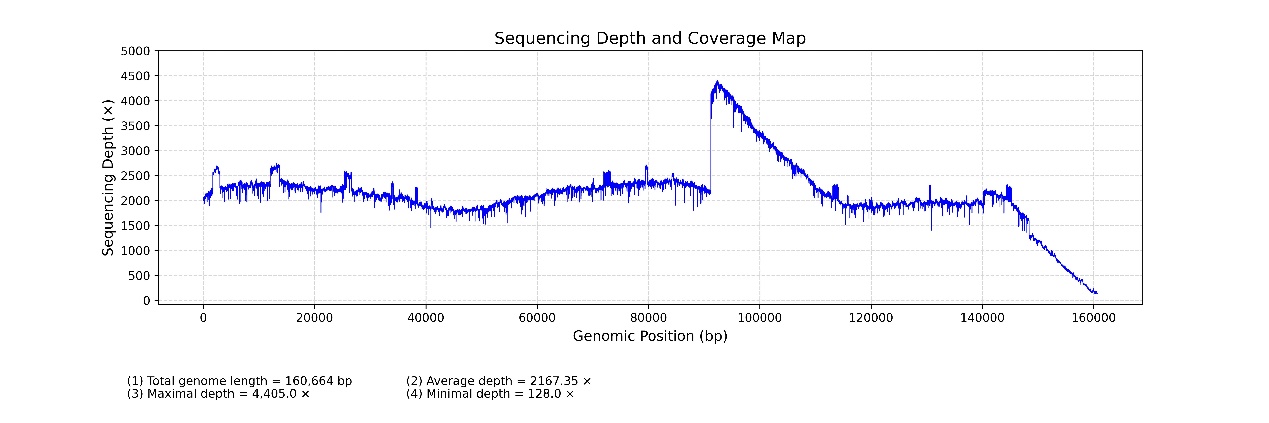
 Figure S1. The read coverage plot of *T. sinensis* chloroplast genome. The x-axis represents the position in the chloroplast genome, while the y-axis shows the sequencing depth.


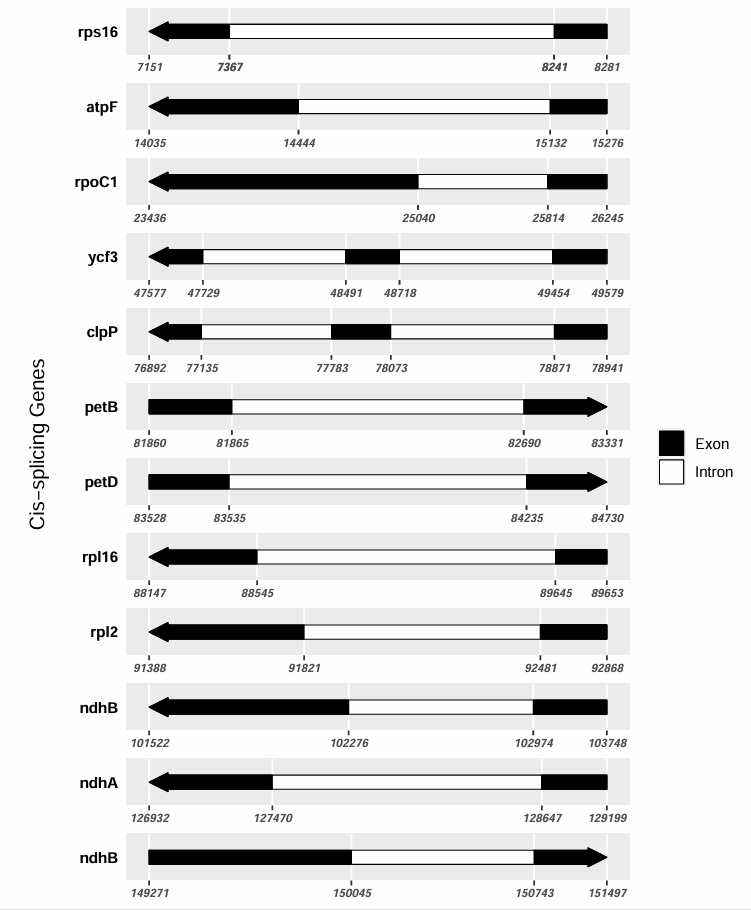


Figure S2. The map of the cis-splicing genes in the *T. sinensis* chloroplast genome. The direction of the each gene is described by the associated arrow. The gene consists of exons (black parts) and introns (white parts). The names of genes are positioned on the left. The numbers below each arrow indicate the corresponding position in the genome.


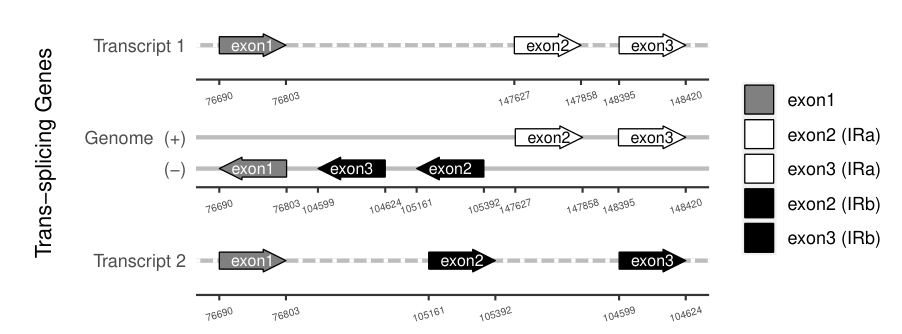


Figure S3. Schematic map of the trans-splicing genes (*rps12*) in the *T.*

*sinensis* chloroplast genome. Exons of the same gene are located in different regions of the genome and are transcribed separately, forming distinct primary transcripts (Transcript 1 and Transcript 2). The exons are then joined together during RNA processing to form a mature mRNA. Exon1 is located in the single-copy region, while exon2 and exon3 are located within the inverted repeats (IRa and IRb). Arrows indicate the transcriptional direction. Exon colors correspond to their genomic locations, with light gray for exon1, white for exon2/exon3 in IRa, and black for exon2/exon3 in IRb. Plus (+) and minus (–) symbols denote the strands of the genome.


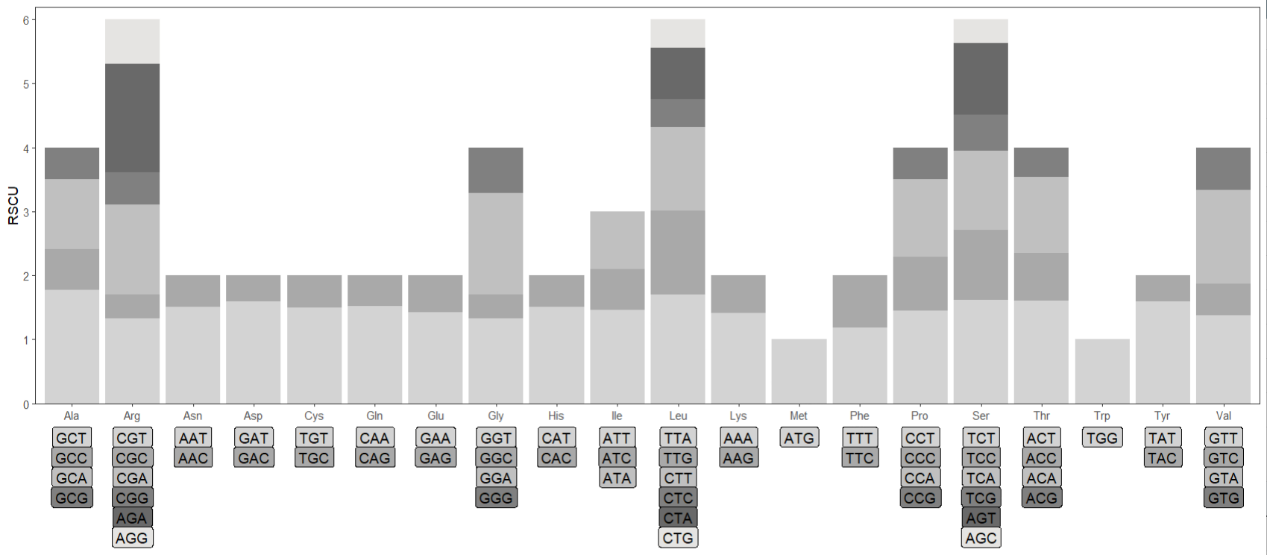


Figure S4. Analysis of codon preference in the chloroplast genome of *T. sinensis*. Colored blocks represent all codons encoding each amino acid, and the height of the upper row represents the sum of the RSCU values of all codons.


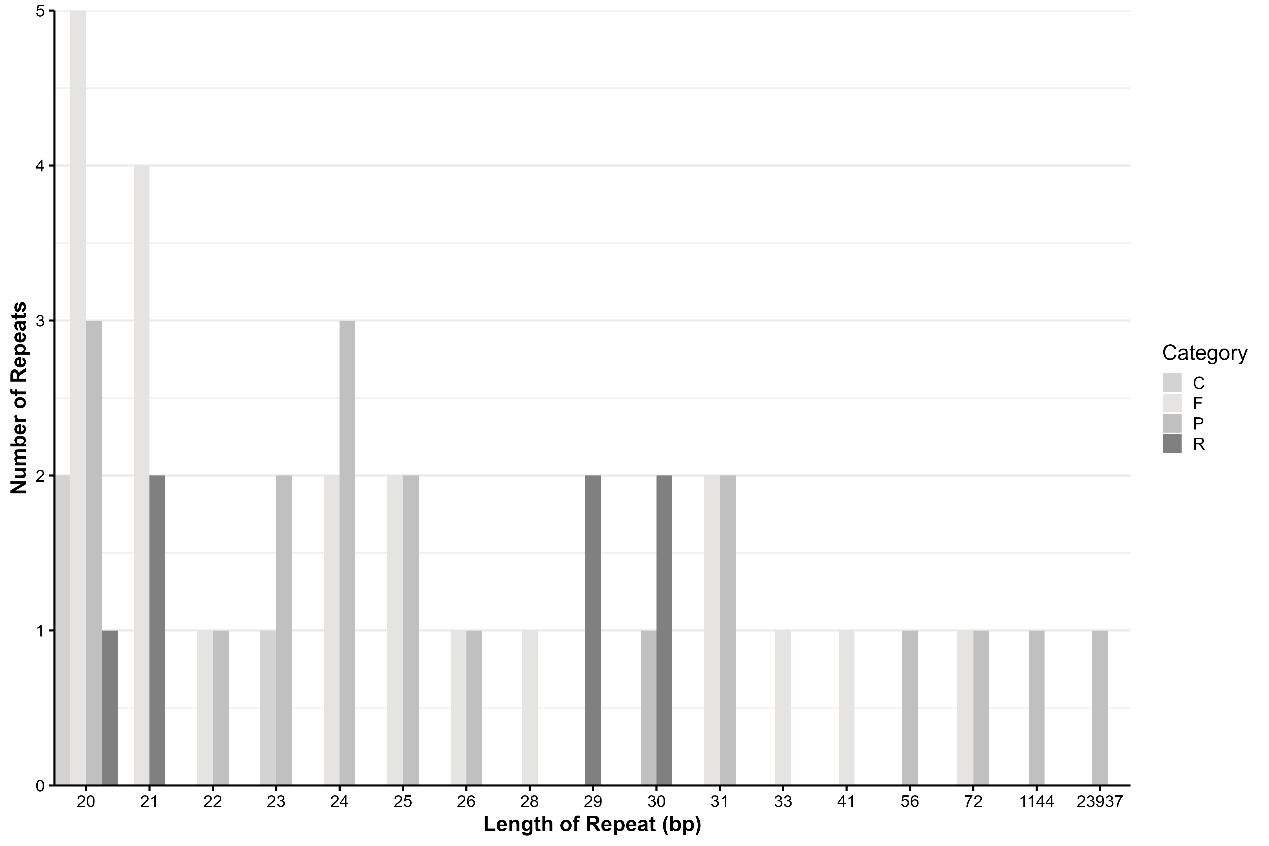


Figure S5. Summary of interspersed repeat sequence. The X-axis is the length of repeat unit.The Y-axis represents the number of repeats having different lengths.F: forward repeat; P: palindrome repeat; R: inverted repeat; and C: complementary repeat.


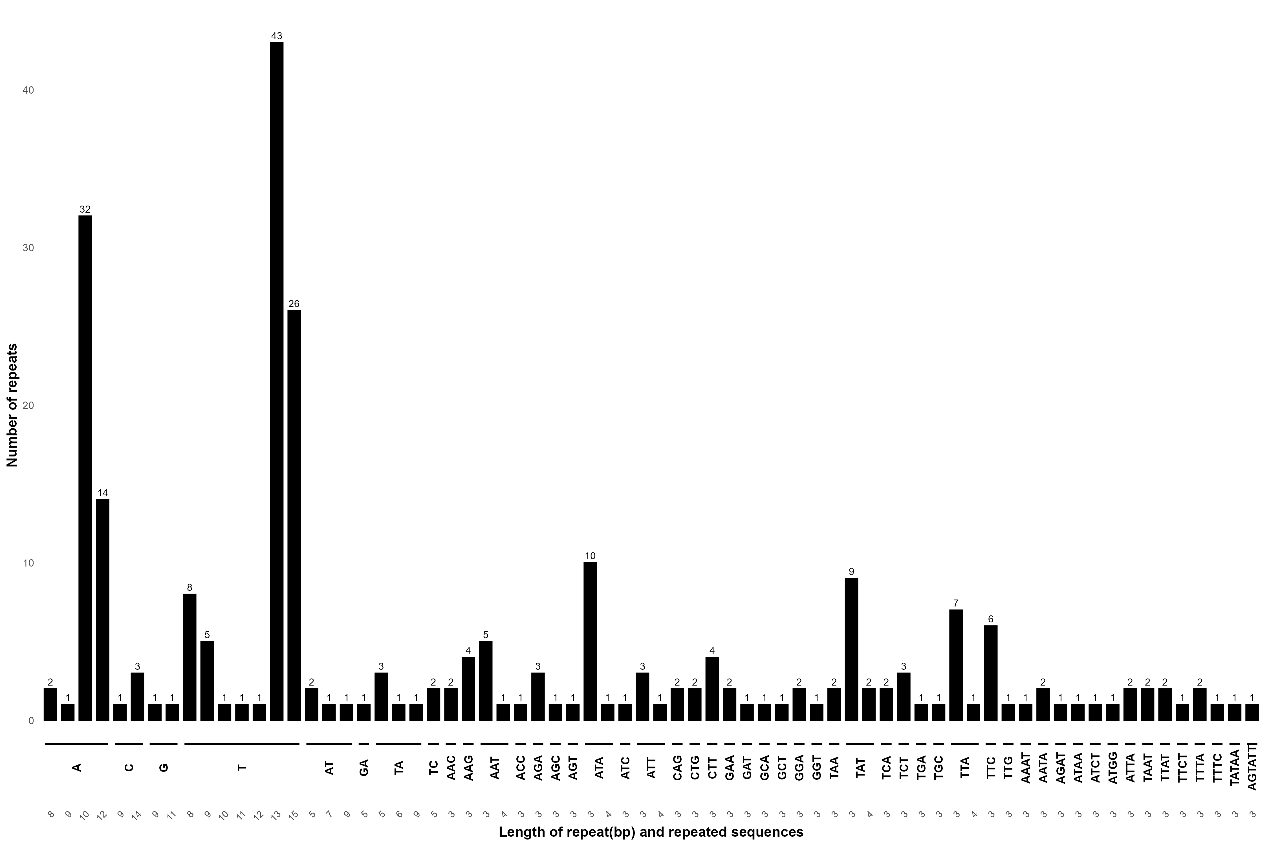


Figure S6. Classification and frequency of SSRs identified in the chloroplast genome of *T. sinensis*. A total of 256 SSRs were detected, including 140 mononucleotide, 12 dinucleotide, 85 trinucleotide, 17 tetranucleotide, one pentanucleotide, and one hexanucleotide repeat. Mononucleotide SSRs were the most abundant, accounting for 54.69% of the total.


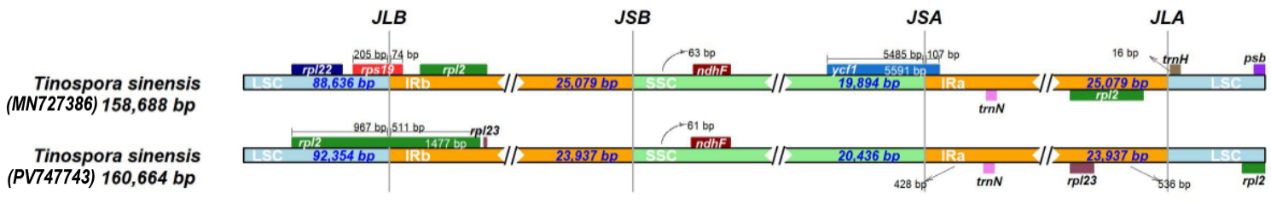


Figure S7. The diagram illustrates the junctions between LSC, SSC, and IR regions in the newly assembled chloroplast genome (PV747734) and the reference genome (MN727386). Gene locations at the IRb/LSC, IRb/SSC, IRa/SSC, and IRa/LSC boundaries are shown, with distances from junction sites indicated. Differences in gene positioning across the two genomes reflect structural variation in the expansion or contraction of IR regions.
